# Plantar mechanical stimulation attenuates protein synthesis decline in disused skeletal muscle via modulation of nitric oxide level

**DOI:** 10.1038/s41598-021-89362-6

**Published:** 2021-05-07

**Authors:** Sergey A. Tyganov, Ekaterina Mochalova, Svetlana Belova, Kristina Sharlo, Sergey Rozhkov, Vitaliy Kalashnikov, Olga Turtikova, Timur Mirzoev, Boris Shenkman

**Affiliations:** Myology Laboratory, Institute of Biomedical Problems RAS, Khoroshevskoe shosse 76a, Moscow, Russian Federation 123007

**Keywords:** Molecular biology, Physiology

## Abstract

Both research conducted under microgravity conditions and ground-based space analog studies have shown that air pump-based plantar mechanical stimulation (PMS) of cutaneous mechanoreceptors of the sole of the foot is able to increase neuromuscular activity in the musculature of the lower limbs. This type of stimulation is able to attenuate unloading-induced skeletal muscle atrophy and impaired muscle function. The aim of the present study was to evaluate the effects of PMS on anabolic signaling pathways in rat soleus muscle following 7-day hindlimb suspension (HS) and to elucidate if the effects of PMS on anabolic processes would be NO-dependent. The soles of the feet were stimulated with a frequency of 1-s inflation/1-s deflation with a total of 20 min followed by 10 min rest. This cycle was repeated for 4 h each day. We observed a decrease in the soleus muscle mass after 7-day HS, which was not prevented by PMS. We also observed a decrease in slow-type fiber cross-sectional area (CSA) by 56%, which significantly exceeded a decrease (–22%) in fast-type fiber CSA. PMS prevented a reduction in slow-twitch fiber CSA, but had no effect on fast-twitch fiber CSA. PMS prevented a 63% decrease in protein synthesis after 7-day HS as well as changes in several key anabolic signaling regulators, such as p70S6k, 4E-BP1, GSK3β, eEF-2, p90RSK. PMS also prevented a decrease in the markers of translational capacity (18S and 28S rRNA, c-myc, 45S pre-rRNA). Some effects of PMS on anabolic signaling were altered due to NO-synthase inhibitor (L-NAME) administration. Thus, PMS is able to partially prevent atrophic processes in rat soleus muscle during 7-day HS, affecting slow-type muscle fibers. This effect is mediated by alterations in anabolic signaling pathways and may depend on NO-synthase activity.

## Introduction

In mammals, a predominantly slow soleus muscle is one of the most active muscles that works 11–15 h per day^1^. Daily mean electromyographic (EMG) amplitudes and integrated EMG levels in the soleus are 2- to threefold higher than in predominantly fast medial gastrocnemius and tibialis anterior muscles^1^. Along with other tonic muscles, the soleus muscle ensures the stability of the mammalian organism in the in the Earth's gravitational field. Its contractile activity is mainly determined by two biomechanical factors: axial loading and ground reaction force. Both factors exert their influence under conditions of Earth's gravity and are eliminated under weightlessness. Support withdrawal (i.e. removal of the ground reaction force) is one of the key factors of spaceflight, which has a significant impact on human motor system. In recent years, the efforts of a number of authors have revealed the neuromuscular mechanisms of support perception and formed the idea of the importance of afferent signals from mechanoreceptors of the soles of the feet^2–7^. In human spaceflight studies, as well as in animal unloading experiments it was found that support withdrawal, among other factors, leads to significant changes in the mechanisms of motor control^8–12^. In particular, mechanical unloading is able to shift the activity pattern of the motor units, inactivating slow motor units and thereby determining a decrease in postural muscle tone^3,13,14^. Skeletal muscle undergoes profound changes in response to unloading conditions: reductions in the rate of protein synthesis and fiber size, increased protein degradation; changes in signaling pathways regulating protein turnover as well as alterations in gene expression, in particular, myosin heavy chain isoforms^15–18^.

Usually, the role of tonic activity in maintaining muscle properties is investigated in experiments with imposed forms of contractile activity, imitating the natural activity pattern of slow postural muscles under unloading. In human and rodent studies, either chronic low-frequency (10–25 Hz) electrostimulation or mechanical stimulation of the mechanoreceptors of the sole of the foot is applied. In dry immersion study (unloading model in which the subject is suspended in water but separated from it by a waterproof material) low-frequency (25 Hz) chronic electrostimulation was able to prevent a decrease in the maximum isometric strength of the extensor muscles of the human leg^19^. In addition, chronic electrical stimulation (10 Hz, 8 h per day) applied during 21-day hindlimb suspension (HS) was able to attenuate unloading-induced decreases in soleus muscle weight and maximum isometric tension^20^. Low-frequency electrostimulation can also prevent a decrease in longitudinal muscle stiffness induced by HS^20^. Daily application of low-frequency electrostimulation (20 Hz, twice a day for 3 h) during 28-day HS can partly attenuate the reductions in fiber diameter and satellite cell activity in rat soleus muscle^21^*.*

Afferent signals from various mechanosensors (Ruffini endings, Merkel discs, Pacinian corpuscles, Meissner’s corpuscles) located in the soles of the feet make a significant contribution to the maintenance of the tonic activity of postural muscles^22–25^. Under the influence of pressure, vibration, stretching, these mechanosensors generate action potentials that produce excitation and/or inhibition of the functionally related pools of motoneurons in spinal cord^24^. In response to this afferent signals postural corrective responses are evoked by calf muscles^25^. Accordingly, mechanical stimulation of the plantar mechanoreceptors under gravitational unloading is able to induce an increase in the tonic activity of the postural muscles. Mechanical plantar stimulation can be obtained in different ways. Passive stimulation during unloading can be elicited by using a fixed immovable platform, which contacts the foot of the hindlimb. Such kind of artificial support was tested in rat study by Nemirovskaya & Shenkman (2002)^26^. In some animal studies vibrostimulation of the foot during unloading conditions has been used^27,28^. The main disadvantage of such kind of stimulation is that vibrostimulation indirectly affects the entire limb of the animal and activates type Ia sensory fibers in muscle spindles. Thus, it is difficult to separate the impact of vibrostimulation on the foot from the impact of vibrostimulation on the cutaneous mechanoreceptors in the sole. As for passive stimulation, it appears to be less efficient as compared to active stimulation. At the same time, in humans and animals it was found that active mechanical stimulation of the soles of the feet (plantar mechanical stimulation, PMS) under mechanical unloading can reduce the development of atrophic changes in postural muscles, e.g. slow-to-fast fiber type shift and degradation of cytoskeletal proteins^3,5–8,12,26,29,30^. It has been revealed that support afferentation plays an important role in the maintaining of structure and function of postural muscles in the gravitational field of the Earth. We can also state that the withdrawal of support afferentation can be one of the main causes for the development of atony and atrophy of postural muscle under conditions of gravitational unloading. At the same time, literature analysis indicates that the molecular mechanisms underlying the maintenance of the structural and contractile characteristics of postural muscles during the PMS under unloading conditions have not been thoroughly studied. In our laboratory, it was previously shown that PMS can prevent a decrease in the rate of global protein synthesis and changes in 4E-BP1, GSK-3β, AMPK phosphorylation in rat soleus muscle under short-term hindlimb suspension^31^. It was also demonstrated that the application of PMS during short-term unloading (1–3 days) can prevent or attenuate an increase in MuRF-1 mRNA expression^31^ , a decrease in MyHC Iβ expression via calcineurin-NFATc1 signaling pathway^32^ as well as the loss of neuronal NO-synthase^33^. It has been also shown that nitric oxide synthase activity is necessary for the PMS-mediated prevention of slow-to-fast muscle fiber-type shift and myosin I and IIa mRNA transcription decreases during 7-day hindlimb unloading^34^. Studies on PMS effects are not merely relevant for basic research but also of importance for applied physiology as possible countermeasure against muscle deconditioning induced by prolonged disuse (spaceflight, bedrest, cast immobilization)^3,6^.

One of the issues in the gravitational control of postural muscles concerns molecular mechanisms that are responsible for the maintaining of muscle proteostasis after PMS. We hypothesized that one of the possible mechanisms could be associated with a change in the level of nitric oxide (NO) in postural soleus muscle. NO is synthesized by the enzyme nitric oxide synthase (NOS)^35^. There are three known isoforms of NOS in mammals: type I neuronal NOS (nNOS), type II inducible NOS (iNOS) and type III endothelial NOS (eNOS)^36,37^. A well-known an antagonist of NOS, L-N^G^-Nitro arginine methyl ester (L-NAME) is able to inhibit both nNOS and eNOS in skeletal muscle^35^. Resistance arteries located in skeletal muscle can serve as a source of NO. Blood flow, which is extremely decreased in lower limbs under unloading conditions^38^, is sensed by the endothelial cells of resistance arteries which can release NO causing vasodilation. But, unlike elastic arteries, for which endothelium-dependent vasodilation occurs mainly through the NO-pathway, other vasodilative signaling pathways may play a role in resistance vessels^38^. It was previously shown that gravitational unloading (rat HS) leads to a significant decrease in the NO levels and nNOS mRNA expression in rat the soleus muscle^39^. These data correspond to other studies. For example, total nNOS content was decreased after prolonged HS^40^, bed-rest and immersion^41^. A decrease in nNOS content in mouse skeletal muscle was also observed under conditions of 90-day spaceflight^42^. Using electron paramagnetic resonance (EPR) spectroscopy, Sharlo and colleagues (2021) have recently showed that 7-day HS results in a significant decrease in NO content in rat soleus compared to control animals, and PMS application during 7-day HS is able to prevent this NO decrease in L-NAME-dependent manner^34^. In the current study, we assumed that if a reduced level of NO in the soleus muscle under unloading conditions promotes the development of atrophic changes^35^, then the maintenance of NO levels after PMS would lead to stabilization of the anabolic signaling pathways. In order to test this assumption, NO synthase inhibitor L-NAME was applied to the group of animals subjected to HS and PMS.

## Materials and methods

### Ethical approval

All procedures with the animals were approved by the Biomedicine Ethics Committee of the Institute of Biomedical Problems of the Russian Academy of Sciences/Physiology section of the Russian Bioethics Committee (protocol no. 421, 14.04.2016). A statement confirming the study was carried out in compliance with the ARRIVE guidelines. All efforts were made to minimize the animals’ pain and suffering. Animals were housed in a temperature-controlled room on a 12:12-h light–dark cycle with food pellets and water provided ad libitum. Wistar male rats were obtained from the certified Nursery for laboratory animals of the Institute of Bioorganic Chemistry of the Russian Academy of Sciences (Pushchino, Moscow region). On completion of the experiments, the animals were sacrificed by i.p. injection of tribromoethanol overdose (750 mg/kg) followed by cervical dislocation.

### Study design

3-month old male Wistar rats of 180–210 g were randomly assigned to 4 groups (n = 8 per group): 1) cage control (C), 2) hindlimb suspension for 7 day (7HS), 3) hindlimb suspension for 7 day + plantar mechanical stimulation (PMS), 4) hindlimb suspension for 7 day + plantar mechanical stimulation + daily L-NAME (nitric oxide synthase inhibitor) intraperitoneal injection (PMS + LN) at a concentration of 50 mg/kg body weight^43^. All other experimental groups received a placebo equivalent in volume. After the experiment, the rats were sacrificed as described above, and their right soleus muscles were dissected, weighed and immediately frozen in liquid nitrogen. All protein and mRNA measurements were obtained from full-muscle tissue.

The experimental design did not include control rats that received L-NAME only. It was shown that L-NAME reduces NO production which results in increased total peripheral resistance and high blood pressure^44^, so profound NO deficiency caused by L-NAME during HS leads to a high probability of stroke, myocardial infarction and congestive heart failure. We also suggest that the effect of L-NAME on unloaded and non-unloaded animals would not be equal, as the unloading-induced changes include not only NO decrease, but also alterations in calcium concentration, myokines, reactive oxygen species (ROS), ATP/ADP ratio, etc., so the consequences of NO modulation under these signaling conditions may vary from NO modulation in non-unloaded soleus. Calcium ions were shown to affect many NO-dependent signaling pathways, for example, calcium can activate calpains, while NO is a potent calpain inhibitor, so NO would have differently inhibit calpains under high calcium or low calcium concentrations. High ROS levels during hindlimb unloading could also cause NO to form peroxynitrite that is able to affect protein turnover via protein oxidation and proteolysis. That is why we are strongly convinced that nitric oxide modulation would lead to different signaling changes in unloaded and non-unloaded soleus. Divergent effects of nitric oxide inhibition with different activity levels were shown earlier^45^, so we consider that this situation might also be relevant to our experiment. Moreover, in rodents, 7-day L-NAME administration can induce the state of hypertension^46^. L-NAME-induced hypertension in control (non-unloaded) animals may lead to severe cardiovascular issues*.* Based on the aforementioned arguments, the C + L-NAME group was not included in the present study.

### Hindlimb suspension

Mechanical unloading was carried out using a standard hindlimb suspension (HS) model^47^. Briefly, a strip of adhesive tape was applied to the animal’s tail, which was suspended by passing the tape through a swivel that was attached to a metal bar on the top of the cage. This allowed the forelimbs to have contact with the grid floor and allowed the animals to move around the cage for free access to food and water. The suspension height was adjusted to prevent the hindlimbs from touching any supporting surface while maintaining a suspension angle of approximately 30°. This model causes atrophy of the postural muscles, and subsequent recovery of the hindlimbs evokes muscle regeneration, resulting in the restoration of muscle mass.

### Plantar mechanical stimulation

**Figure 1:**
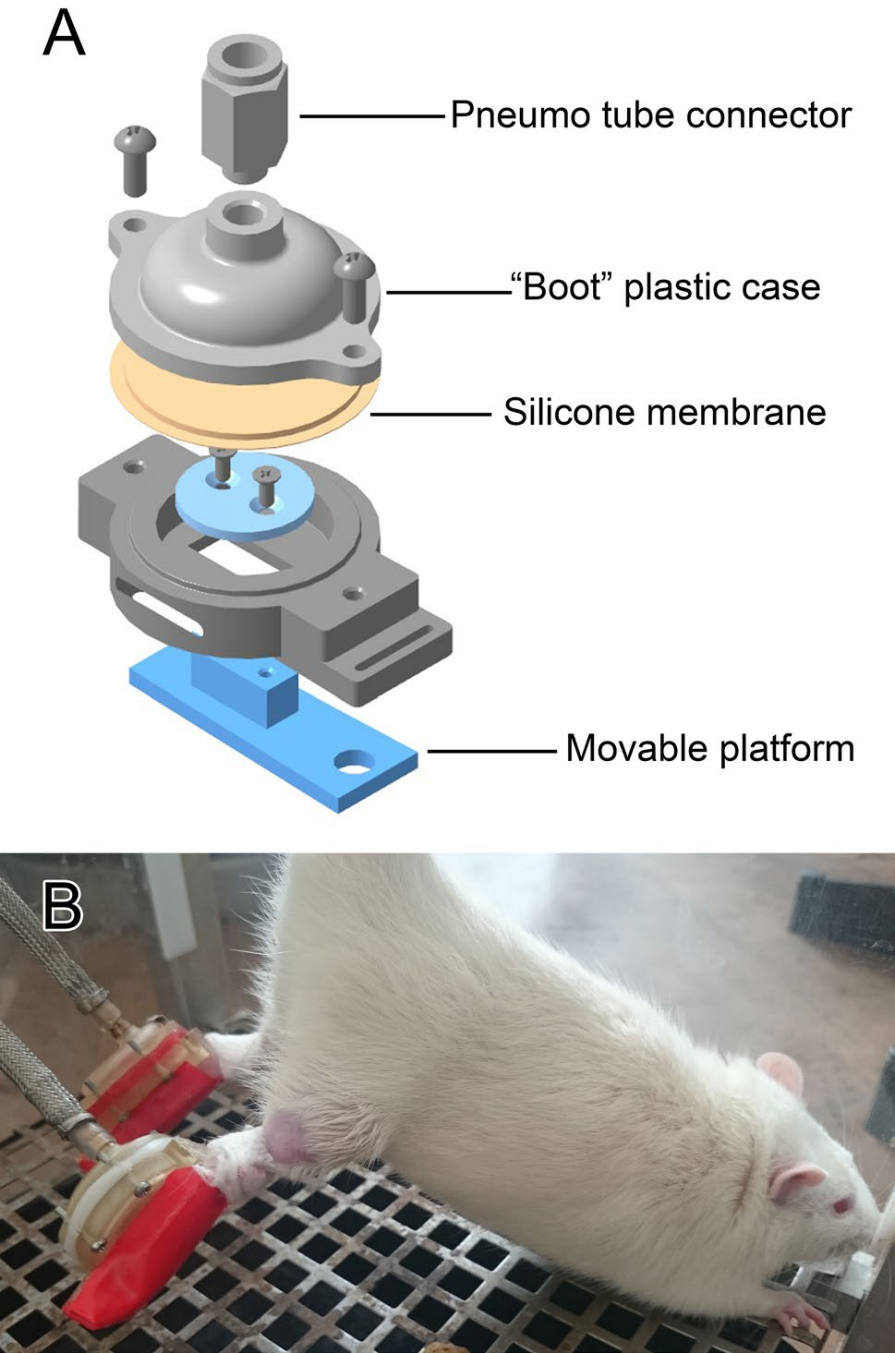
(**A**) Schematic image of the “boot” for plantar stimulation of the rat soles. (**B**) Tail-suspended rat with attached “boot”.

Plantar mechanical stimulation was performed by using a model previously described by Kyparos et al. with modifications^5,32^. Plantar stimulation was applied using plastic 3D-printed “boot” with a movable platform inside that allows regulating pressure and frequency of stimulation of the sole (Fig. 1 A, B). This apparatus was attached to the animal foot above the ankles using the adhesive patch. Pressure was applied to the foot by a movable platform contacting the sole of the foot using an electronically controlled air-pump attached to a hose. Each sole was stimulated with a frequency of 1-s inflation /1-s deflation with a total of 20 min followed by 10 min rest. This cycle was repeated 8 times within 4 h each day of HS. The 1-s inflation/1-s deflation cycle was used to simulate rat’s walking pattern. The apparatus was removed after completion of all cycles.

### Western blot analysis

Western blot analysis and total protein fraction extraction was performed as described previously^48^. Following primary antibodies (diluted in PBS-T) was used in experiment: p-p70S6K (Thr 389) (1:500; Santa Cruz Biotechnology, USA, sc-11759) and p70s6k (1:1000, Cell Signaling Technology, USA, #9202), p-4E-BP1 (Thr37/46) (1:1000, Cell Signaling Technology, USA, #2855) and 4E-BP-1 (1:2000, Cell Signaling Technology, USA, #9452), p-GSK-3β (Ser 9) (1:1000, Cell Signaling Technology, USA, #9322) and GSK-3β (1:1000, Cell Signaling Technology, USA, #12,456), p-eEF2 (Thr56) (1:1000, Cell Signaling, USA, #2331) and t-eEF2 (1:2000, Cell Signaling Technology, USA, #2332), p-AKT (Ser473) (1:1000, Cell Signaling Technology, USA, #4058) and AKT (1:2000, Cell Signaling Technology, USA, #9272), puromycin (1:3000, Kerafast Inc., Boston, USA, EQ0001), GAPDH (1:10,000, Applied Biological Materials Inc., Richmond, British Columbia, Canada, no. G041), p-IRS-1 (Ser639) (1:1000, ThermoFisher, USA, PA5-37,613) and IRS-1 (1:1000, Cell Signaling Technology, USA, #2390), p-p90RSK (Ser380) (1:1000, Cell Signaling, USA, #9341) and RSK1 (1:2000, Cell Signaling Technology, #8408). The membranes were incubated with horseradish peroxidase-conjugated secondary antibodies to rabbit (1:30,000; Jackson Immuno Research, USA, #111–035-003) or mouse (diluted 1:20,000; Bio-Rad Laboratories, CA, USA, # 1,706,516) immunoglobulins. The protein bands were quantified using C-DiGit Blot Scanner (LI-COR Biotechnology, USA) and Image Studio Digits 4.0.21 software. Following image capture of phosphorylated proteins, membranes were stripped of the phosphospecific antibodies, using RestoreTM Western Blot Stripping Buffer (Thermo Scientific, USA), for 30 min at 37 °C after which the membranes were re-probed with primary antibodies for each respective total protein. The signal from the phospho-protein was normalized to the total protein. For protein synthesis detection, the measurements of the chemiluminescent signals were performed by determining the density of each whole lane with the entire molecular weight range of puromycin-labeled peptides. Each gel contained samples from the all groups. Protein samples were run at least in duplicate on the same gel. The representative blots are of the same samples (phospho and total). GAPDH content was used as loading control.

### SUnSET technique for measuring the rate of protein synthesis

SUnSET technique uses standard Western blotting and immunohistochemical technologies to visualize and quantify the rates of protein synthesis^49,50^. For measurements of protein synthesis, rats were given an intraperitoneal injection of 0.04 μmol/g puromycin hydrochloride (Enzo Life Sciences, NY, USA) dissolved in PBS. At exactly 30 min after injection, muscle tissue was extracted and frozen in liquid nitrogen for WB analysis.

### RNA isolation and electrophoresis

RNA isolation and electrophoresis were performed as previously described^31^. The samples of muscle tissue were sliced using cryostat (Leica, Germany) and weighed on electronic laboratory balance. Total RNA was extracted from frozen soleus muscle samples using RNeasy Micro Kit (Qiagen, Germany) according to the manufacturer’s protocol. RNA concentration was analyzed at 260 nm. RNA quality of purification was evaluated according to 260/280 and 260/230 ratios. The electrophoresis was carried out in 1.2% agarose gel with ethidium bromide staining in TBE buffer. The total mRNA sample value for electrophoretic gel was evaluated by normalization of the sum value of RNA extracted from the tissue sample to a tissue sample weight. All the samples were mixed with equal value of denaturing buffer (Thermo Scientific) and heated for 70 ºC for 10 min. RiboRuler markers (Thermo Scientific) were used for RNA molecular weight analysis. The results of electrophoresis were analyzed by Gel Doc EZ Imager (Biorad) and Image Studio Digits v. 4.0. software. The rest of RNA solutions were stored at −85 °C and until further RT-PCR procedures. The RNA integrity was assessed by evaluating 28S/18S ratio.

### RT-qPCR analysis

Reverse transcription was performed by incubation of 0.5 μg of RNA, random hexamers d(N)6, dNTPs, RNase inhibitor and MMLV reverse transcriptase for 60 min at 42 °C. The samples to be compared were run under similar conditions (template amounts, duration of PCR cycles). The annealing temperature was based on the PCR primers’ optimal annealing temperature. PCR primers used for RNA analysis are shown in Table 1. The amplification was realtime monitored using SYBR Green I dye and the iQ5 Multicolor Real-Time PCR Detection System (Bio-Rad Laboratories, USA). To confirm the amplification specificity, the PCR products from each primer pair were subjected to a melting curve analysis, and sequencing of the products was provided at least once. Relative quantification was performed based on the threshold cycle (CT value) for each of the PCR samples^51^. RPL19 mRNA was not significantly altered in any of the experimental groups compared to control so it was chosen for the normalization of all quantitative PCR analysis experiments in the current study.

**Table 1:** Primers used for RT-qPCR.

Gene description	Primer sequence
*c-myc*	5′-TTGATGGGGATGACCCTGAC-3' 5′-CTCGCCCAAATCCTGTACCT-3'
*RPL19*	5′-GTACCCTTCCTCTTCCCTATGC-3' 5′-CAATGCCAACTCTCGTCAACAG-3'
*eef2*	5′-GTCCCCAAACAAGCACAACAGG-3' 5′-GGCTTCAGCAACATCCCACTCA-3'
*Pre-45S-rRNA*	5′-TGGGGCAGCTTTATGACAAC-3' 5′-TAGCACCAAACGGGAAAACC-3'

### Determination of the size of slow and fast muscle fibers

Cross-sections from the mid-belly of the muscles were cut at 10 µm in a cryostat (Leica Microsystems, Germany) maintained at –20 °C. The sections were then warmed to room temperature for 15 min and rehydrated by incubating in PBS for 20 min. The rehydrated sections were then incubated for 1 h at 37 °C with primary antibodies against slow or fast myosin heavy chains (MyHC I (slow), 1:400, Sigma, USA, M8421 and MyHC II (fast), Sigma, USA, 1:400, M4276). After washing with PBS, the sections were incubated with the appropriate fluorophore-conjugated secondary antibodies (goat anti-mouse secondary antibody, Alexa Fluor 488, 1:500, Molecular probes, USA, A-11001) for 40 min in the dark at room temperature. After washing in PBS, the stained sections were mounted using the mounting medium (Vector Laboratories) for microscopic analysis.

The sections were examined and photographed using a Leica Q500MC fluorescence microscope with an integrated digital camera (TCM 300F, Leica, Germany), 20× magnification. Image analysis was performed using ImageJ 1.52a software. At least 150 fibers were analyzed in each muscle sample.

### Statistical analysis

All western blot and immunohistochemistry data are shown as mean ± SEM. The means of all groups are shown as % of the control group. mRNA and rRNA data are shown as median and interquartile range (0.25–0.75) ± the minimum and the maximum. To check whether the differences among groups were statistically significant, given the small sample sizes and comparisons between the four groups, the Kruskal–Wallis nonparametric test with Dunn’s multiple range test were applied. A p value less than 0.05 was regarded as statistically significant.

## Results

### Body weight and soleus muscle weight

The weight of rats significantly decreased by 12% (*p* < 0.01) in 7HS and PMS + LN groups compared to the control animals. Soleus muscle mass, normalized to body weight, significantly decreased by 25% (*p* < 0.001) after 7-day HS. In the groups with plantar stimulation (PMS and PMS + LN), a similar decrease was observed (Table 2).

**Table 2:** Body weight and soleus muscle weight of experimental animals.

Group	Body weight before HS, g	Body weight, g	Soleus weight, mg	Body weight/soleus weight, mg/g
*C*	209.4 ± 8.3	221.7 ± 2.8	98.1 ± 2.7	0.44 ± 0.011
*7HS*	204.4 ± 7.5	196.1 ± 5.2*	66.5 ± 2.7*	0.34 ± 0.013*
*PMS*	219.6 ± 3.3	217.4 ± 4.2	71.3 ± 2.4*	0.33 ± 0.014*
*PMS* + *LN*	206.9 ± 7.1	197.7 ± 6.5*	64.1 ± 4.5*	0.33 ± 0.013*

Values are means ± SE. n, number of rats; C, vivarium control group; 7HS, hindlimb suspension for 7 days; PMS, hindlimb suspension for 7 days + plantar mechanical stimulation; LN, hindlimb suspension for 7 days + plantar mechanical stimulation + L-NAME injection.

*Significant difference from C.

### Cross-sectional area of the slow and fast muscle fibers

**Figure 2:**
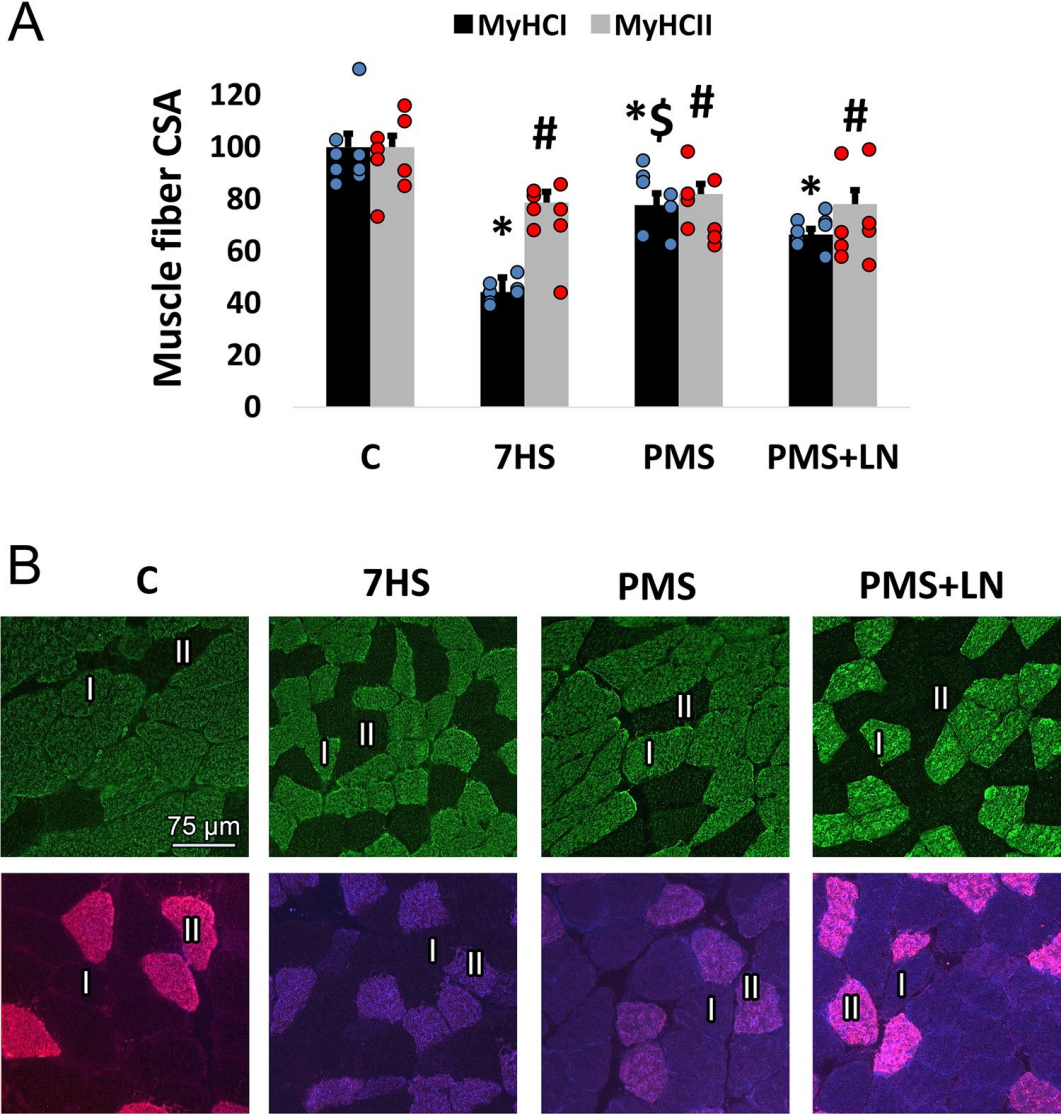
(**A**) Cross-sectional area of slow-type and fast-type fibres in the soleus muscle. (**B**) Representative images showing soleus muscle fibres expressing MyHC I and MyHC II. I—fibres expressing MyHC I; II—fibres expressing MyHC II. Control group (C); 7-days hindlimb-suspended group (7HS); 7-days hindlimb suspended group with plantar mechanical stimulation (PMS); 7-day hindlimb suspended group with plantar mechanical stimulation + L-NAME injection (PMS + LN). Data shown as % of control group. *Significant difference from control group for MyHC I. ^#^Significant difference from control group for MyHC II. ^$^Significant difference from 7HS (*p* < 0.05). Blue and red dots correspond to the number of samples. The cross-sectional area of slow-type and fast-type fibres was analyzed using ImageJ 1.52a software (https://imagej.nih.gov/ij).

The cross-sectional area (CSA) of the soleus muscle fast fibers (MyHC II) significantly decreased by 22% (*p* < 0.01) after 7-day HS. In plantar stimulation groups (PMS and PMS + LN), a similar decrease was observed (Fig. 2A, B). At the same time, the CSA of slow fibers (MyHC I) significantly decreased by 56% (*p* < 0.01) after 7-day HS. Plantar stimulation almost completely prevented this decline in an L-NAME-dependent manner (Fig. 2A, B).

### Anabolic response of the rat soleus muscle

**Figure 3:**
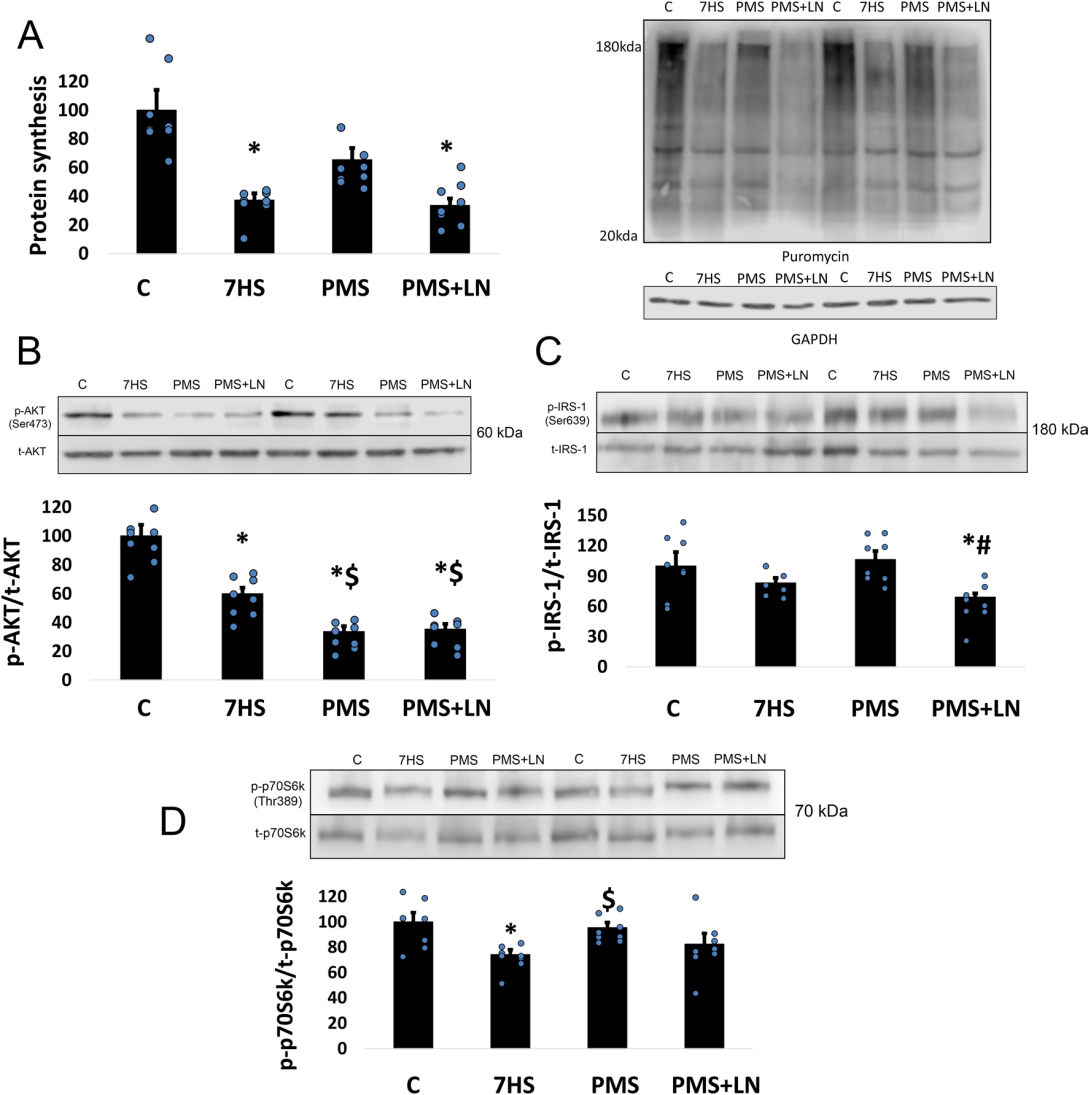
(**A**) Quantification of protein synthesis level. (**B**) Quantification of phospho-AKT (Ser473)/total AKT ratio. (**C**) Quantification of phospho-IRS-1 (Ser639)/total IRS-1 ratio. (**D**) Quantification of phospho-p70S6k (Thr389)/total p70S6k ratio. Control group (**C**); 7-days hindlimb-suspended group (7HS). 7-days hindlimb suspended group with plantar mechanical stimulation (PMS); 7-day hindlimb suspended group with plantar mechanical stimulation + L-NAME injection (PMS + LN). Data shown as % of control group. *Significant difference from control group. ^#^Significant difference from PMS. ^$^Significant difference from 7HS (*p* < 0.05). Blue dots correspond to the number of samples. The quantification of the proteins was performed using Image Studio Digits 4.0.21 software (https://www.licor.com/bio/image-studio/).

**Figure 4:**
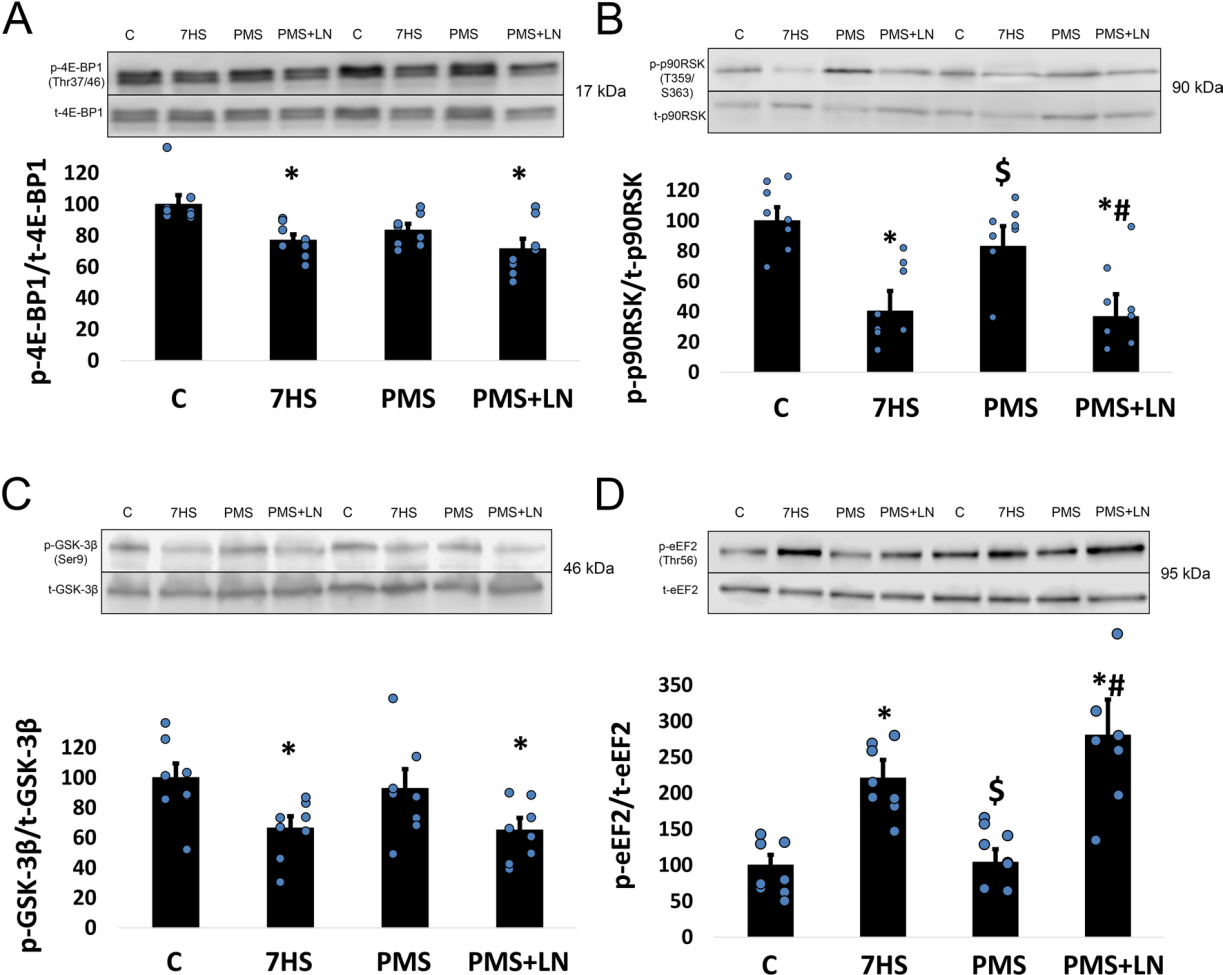
(**A**) Quantification of phospho-4E-BP1 (Thr37/46)/total 4E-BP1 ratio. (**B**) Quantification of phospho-p90RSK (Ser380)/total p90RSK ratio. (**C**) Quantification of phospho-GSK-3β (Ser9)/total GSK-3β ratio. (**D**) Quantification of phospho-eEF2 (Thr56)/total eEF2 ratio. 7-days hindlimb suspended group with plantar mechanical stimulation (PMS); 7-day hindlimb suspended group with plantar mechanical stimulation + L-NAME injection (PMS + LN). Data shown as % of control group. *Significant difference from control group. ^#^Significant difference from PMS. ^$^Significant difference from 7HS (*p* < 0.05). Blue dots correspond to the number of samples. The quantification of the proteins was performed using Image Studio Digits 4.0.21 software (https://www.licor.com/bio/image-studio/).

The rate of protein synthesis measured by the SUnSET method significantly decreased by 63% (*p* < 0.05) after 7 days of HS compared to the control group. PMS attenuated 7-day unloading-induced decrease in protein synthesis, as we observed non-significant 35% reduction in the PMS group compared to the control group. However, L-NAME treatment abolished the effect of PMS on muscle protein synthesis (Fig. 3A). IGF1-Akt/PKB/mTORC1 is a canonical signaling pathway that plays a key role in the regulation of protein synthesis. The initial activation of this signaling pathway occurs through the binding of insulin-like growth factor 1 (IGF-1) to its specific receptor (IGF-1R), which triggers a signaling cascade leading to the phosphorylation of insulin receptor substrate-1 (IRS-1). Seven-day HS led to 31% (*p* < 0.05) decrease in IRS-1 Ser 639 phosphorylation only in the group with support stimulation and L-NAME injection (Fig. 3C). Mammalian/mechanistic target of rapamycin complex 1 (mTORC1), a key complex controlling protein synthesis, is activated by protein kinase B (AKT), which phosphorylates and inactivates tuberous sclerosis complex 2 (TSC2), an endogenous mTORC1 inhibitor. AKT activation is carried out by phosphorylation of two amino acid residues Thr308 и Ser473. The content of phosphorylated form of AKT significantly decreased after 7 days of HS, and in both PMS groups a further decrease in AKT phosphorylation was observed (Fig. 3B). Ribosomal protein S6 kinase p70 (p70S6k) and eukaryotic translation initiation factor 4E-binding protein 1 (4E-BP1) are well-known downstream targets of mTORC1^52^. 7-day HS resulted in a 26% (*p* < 0.05) decrease in p70S6k phosphorylation compared to the control group, and PMS was able to prevent this decline (Fig. 3D). PMS also prevented an unloading-induced decrease in 4E-BP1 phosphorylation (Fig. 4A). The phosphorylation status of ribosomal kinase p90RSK (a marker of ERK1/2-signaling pathway) significantly decreased by 60% (*p* < 0.05) after 7-day HS as compared with the control values (Fig. 4B). However, p90RSK phosphorylation did not differ from the control levels in the PMS group. L-NAME administration abolished the effect of PMS on p90RSK phosphorylation (Fig. 4B). In addition to the regulation of mTORC1-signaling, AKT is also able to phosphorylate glycogen synthase kinase 3β (GSK-3β) on Ser9, which can lead to the activation of mRNA translation initiation via eukaryotic translation initiation factor 2B (eIF2B). Also, GSK-3β is able to phosphorylate an important cytoskeletal protein desmin and the component of focal contacts β-catenin, which subsequently leads to the degradation of these proteins by proteasome^53,54^. PMS was able to prevent the unloading-induced reduction (−34%, *p* < 0.05) in GSK-3β (Ser9) phosphorylation, however in the PMS + LN group GSK-3β phosphorylation was significantly lower than in the control group (Fig. 4C).

**Figure 5:**
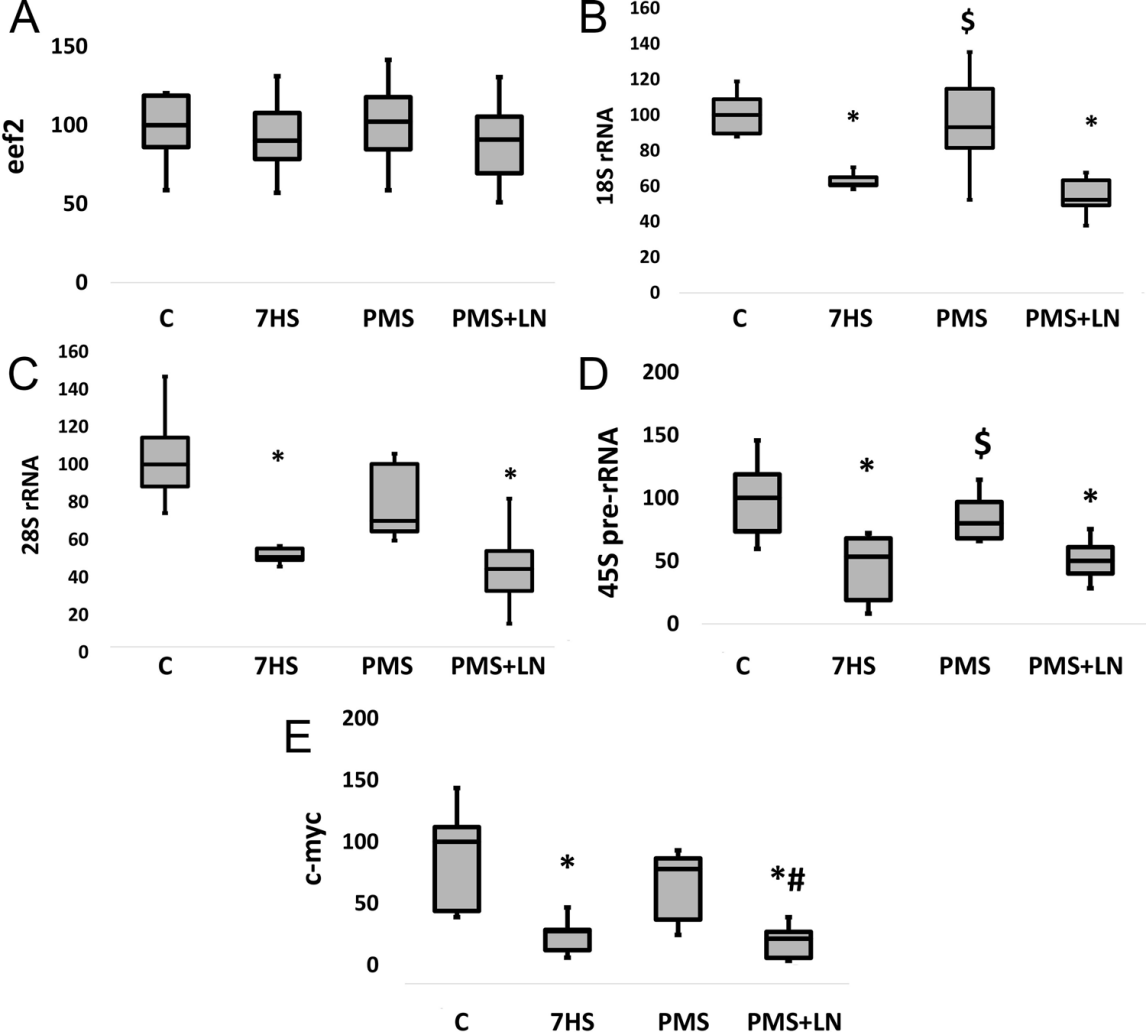
(**A**) eEF2 mRNA content. (**B**) 18S rRNA content. (**C**) 28S rRNA content. (**D**) 45S pre-rRNA content. (**E**) c-myc mRNA content. Control group (**C**); 7-days hindlimb-suspended group (7HS); 7-days hindlimb suspended group with plantar mechanical stimulation (PMS); 7-day hindlimb suspended group with plantar mechanical stimulation + L-NAME injection (PMS + LN). Data shown as % of control group. *Significant difference from control group. ^#^Significant difference from PMS. ^$^Significant difference from 7HS (*p* < 0.05). Blue dots correspond to the number of samples.

The rate of protein synthesis is also dependent on the mechanisms regulating mRNA translation elongation^55^. Our study showed that 7-day HS resulted in a significant increase (+ 121%) (*p* < 0.01) in the inhibitory phosphorylation of eukaryotic elongation factor 2 (eEF2) (Thr56) compared to the control group. The application of PMS during unloading prevented the increase in eEF2 phosphorylation and L-NAME administration cancelled the effect of PMS (Fig. 4D). At the same time, there were no differences in eEF2 mRNA expression across the groups (Fig. 5A).

As for the markers of ribosome biogenesis (translational capacity), PMS prevented a 1.67- and twofold decrease in the content of 18S and 28S rRNA, respectively, after 7-day HS in L-NAME- dependent manner (Fig. 5B, C). 45S pre-rRNA (18S and 28S rRNA precursor) had a similar pattern of expression (Fig. 5D) with a 1.85-fold decrease in the 7HS group and no significant changes in PMS group relative to the control values. Another marker of ribosome biogenesis, transcription factor c-myc, is known to be involved in the regulation of RNA polymerase I activity^56^ and proteins that control rRNA processing^57^. Unloading for 7 days resulted in a significant 2.6-fold (*p* < 0.05) decrease in c-myc mRNA expression compared to the control values. PMS prevented unloading-induced downregulation of c-myc expression in the L-NAME-dependent manner (Fig. 5E).

## Discussion

The present study tested the hypothesis that 4-h PMS performed during 7-day HS would attenuate unloading-induced suppression of anabolic processes in rat soleus muscle via NO-dependent mechanism. The study provides several novel findings:

PMS attenuated a decline in slow-twitch fiber CSA but did not affect the size of fast-twitch muscle fibers in rat soleus muscle after 7-day HS.

PMS attenuated protein synthesis decline in rat soleus after 7-day HS.

Effects of PMS on muscle protein synthesis were accompanied by alterations in the markers of both translational capacity (18S + 28S rRNA; 45S-pre rRNA, c-myc) and translational efficiency (p70S6k, p90RSK, eEF2).

Effects of PMS on some anabolic parameters were mediated via NO-dependent mechanism.

Support afferentation facilitates tonic activity of small motoneurons that control the activity of slow muscle fibers^2,29^. In the studies by Miller et al.^58^ and Vinogradova et al.^59^ it was shown that PMS in humans during unloading (dry immersion) prevented a decrease in the electrical, and therefore mechanical activity of the postural soleus muscle. In the present study, one-week HS resulted in a significant reduction in the CSA of both slow and fast muscle fibers; however the reduction in the CSA was greater in slow fibers than in fast fibers. This result is in a good agreement with previously published data^60^. Skeletal muscles that express predominantly slow-type isoform of MyHC appear to be more sensitive to unloading stimuli in comparison to skeletal muscles expressing primarily fast-type isoforms of MHC^60^. In line with Kyparos et al.^5^, PMS in the present study significantly attenuated atrophic changes in slow but not fast muscle fibers. Similar data were earlier obtained in our laboratory in human dry-immersion studies^7,30,33^. We consider several possible reasons for the distinct effect of PMS on different fiber types. It is known that plantar stimulation is able to attenuate a decrease in soleus muscle EMG activity^2,4,12,61^. Such neuromuscular activation of the soleus muscle during unloading (unloaded tonic contractions of the muscle) is apparently sufficient to prevent atrophy of the slow-twitch muscle fibers. On the contrary, mechanical activation of fast motor units may require resistance exercise-type loading since the activation threshold of fast motorneurons is higher than that of slow motorneurons^60,62^. Another explanation is based on considerations provided by Kyparos et al.^5^. There is evidence that some plantar mechanosensors (Merkel disks and Meissner corpuscles) are located near the surface of the skin, whereas Ruffini endings and Pacinian corpuscles are found in the deeper layers of the skin^5,63^. Mechanical stimulation of Ruffini endings and Pacinian corpuscles, which are responsible for the activation of the fast motor units, requires more pressure on the plantar surface^63^.

Our research group has recently analyzed the effect of PMS during 7-day HS on fibre type distribution in rat postural muscle^34^. It has been demonstrated that PMS for 4 h per day during the course of one-week HS is able to prevent slow-to-fast fibre type transition in rat soleus muscle in an NO-dependent manner^34^. This effect of PMS on HS-induced fibre type transitions was associated with a significant NOS/NO-dependent effect of PMS on the mRNA expression of both MyHC I (slow isoform) and MyHC IIa (fast isoform)^34^. Given that basal rates of protein synthesis^50^ as well as the rate of protein synthesis under mechanical unloading or food deprivation may vary in different fibre types within the same muscle , it is possible that fibre type alterations in the present study could affect protein turnover signaling in the present study. Moreover, changes in fibre type distribution could also affect such variables as shortening velocity, oxidative capacity, fatigue resistance, etc. Future studies will be needed to define possible effects of the PMS-induced changes in the distribution of slow and fast fibre types on both protein turnover signaling and mechanical properties of postural skeletal muscles.

In the present study, PMS was able to attenuate the unloading-induced decrease in the global rate of protein synthesis and affect the phosphorylation status of the key signaling proteins of mTORC1-dependent and mTORC1-independent pathways. A significant decrease in muscle protein synthesis in rat soleus muscle following 7-day HS was previously shown in both our laboratory^64^ and others^65,66^. PMS-induced increase in the rate of protein synthesis was most likely associated with the activity of NOS, since the administration of L-NAME (NOS inhibitor) abrogated the effect of PMS on puromycin-labeled peptides. One of the potential NO-dependent mechanisms that could impact protein synthesis may be related to the effect of NO on the GSK3β/eIF2B signaling pathway, and hence mRNA translation initiation. In mouse cultured myotubes it was shown that NO can mediate inhibitory GSK3β (Ser9) phosphorylation^67^. A decrease in GSK3β (Ser9) phosphorylation would lead to the inhibition of eIF2B activity, that has been shown in skeletal muscle both in vivo an in vitro^68,69^. After 7 days of HS, we observed a significant decrease in GSK3β (Ser9) phosphorylation in the rat soleus muscle, which is in line with our earlier published research^64^. In agreement with our previous early unloading study (1–3 days), PMS during 7-day HS prevented a decline in GSK3β phosphorylation, and this effect was abolished by L-NAME administration. A similar response to 7-day unloading and PMS was observed for phospho-p90RSK, a marker of the Ras/Raf/MEK/ERK signaling pathway. In our laboratory, it was previously shown that 3-day HS can lead to reduced p90RSK phosphorylation in rat soleus muscle^70,71^. At the same time, existing data on p90RSK phosphorylation after longer periods of HS are contradictory^64^. At this stage, it is not clear by what mechanism PMS would influence p90RSK phosphorylation and how it is connected to NO.

The mTORC1 protein complex plays a key role in the regulation of translational efficiency^72,73^. We found that PMS did not affect regulatory proteins functioning upstream of mTORC1 (IRS-1, AKT). However, an increase in the level of muscle activity induced by PMS prevented a decrease in the phosphorylation of the mTORC1 substrates, 4E-BP1 and p70S6k. Moreover, this PMS effect was changed by L-NAME administration and therefore was mediated by NO. The effect of PMS could be associated with the mechanosensory functions of mTORC1, i.e. with its ability to perceive and process mechanical signals^74^. In our laboratory, an impairment of transmission of a mechanical signal to mTORC1 in rat soleus was demonstrated after 7 days of HS^48^. It could be linked to a malfunction of various mechanosensors (such as stretch-activated ion channels^48^) as well as the state of fiber cytoskeleton which plays an important role in cell mechanotransduction^75^. It is well-known that unloading is accompanied by a significant decrease in the content of cytoskeletal proteins such as titin, nebulin, desmin^76–78^. The breakdown of these cytoskeletal proteins is often associated with proteolytic enzyme μ-calpain^79^, which can be inhibited by NO^80^. It was previously shown that PMS during dry-immersion is able to prevent a decrease in giant cytoskeletal proteins (titin, nebulin)^8^, as well as NOS content in human skeletal muscle^33^. Thus, PMS-induced muscle activity could lead to the inhibition of µ-calpain and preservation of mechanotransduction to mTORC1.

Inhibitory phosphorylation of eEF2 (Thr56), that controls elongation of amino acid chain on ribosome, is regulated via Ca-calmodulin-dependent activation of eEF2 kinase (eEF2k). eEF2k, in turn, can be inhibited or activated through various signaling events: concentration of calcium ions, pH level, protein kinase A (PKA) and AMP-activated protein kinase (AMPK) activity^81–83^. In our laboratory it was previously shown that HS for 2 weeks leads to a significant increase in eEF2 (Thr56) phosphorylation in rat soleus muscle^84^. In the present study, we observed a significant increase in the level of eEF2 (Thr56) phosphorylation after 7-day HS that was attenuated by PMS. Similar effect of PMS was previously shown after 1 and 3 days of HS^31^. The most studied regulators of eEF2k activity are AMPK and calcium-calmodulin complex. Since AMPK phosphorylation (Thr172) in rat soleus muscle after 7-day HS was found not to differ from the control levels^64^, it is unlikely that increased eEF2 phosphorylation in the present study is AMPK-dependent. On the contrary, it was shown that 7-day HS can induce a significant elevation in cytosolic free calcium concentration in murine soleus muscle^85^. Therefore, calcium-calmodulin complexes could possibly promote the phosphorylation of eEF2 by eEF2k leading to the inhibition of peptide chain elongation.

The rate of protein synthesis is also dependent on translational capacity, the primary determinant of which is ribosome biogenesis. Proto-oncogene c-myc is known to activate RNA polymerase I transcription^56^ and enhance the expression of numerous genes regulating ribosome biogenesis^57^. The unloading-induced decrease in c-myc expression and NOS-mediated protective effects of PMS that were observed in the present study could be associated with a change in the activity of the Wnt/GSK3β/β-catenin signaling pathway^86^. It was shown that β-catenin nuclear translocation can cause activation of c-myc transcription^87–90^. GSK3β is able to phosphorylate β-catenin on key N-terminal serine and threonine residues, thereby marking it for degradation via the ubiquitin–proteasome pathway^91^. At the same time, NO can negatively regulate the activity of GSK3β in skeletal muscle^43,67^. Thus, L-NAME administration could affect GSK3β activity and subsequently attenuate β-catenin degradation resulting in the activation of c-myc mRNA expression.

RNA polymerase I is responsible for the transcription of 45S pre-rRNA, which is subsequently cleaved into 18S, 5.8S, and 28S rRNAs^92–94^. In the present study, changes in 45S pre-rRNA expression across the study groups were similar to that of c-myc. It was earlier shown that HS for 6 or 7 days results in a downregulation of 18S and 28S rRNAs in rat soleus muscle^64,95^. In the present study, unlike our previous 1- and 3-day unloading study^31^, we observed a positive role of PMS in the maintenance of the amount of 18S and 28S rRNAs during 7-day HS. We speculate that the volume/duration of the PMS applied during 1- or 3-day HS probably was not enough to attenuate the decrease in the markers of ribosome biogenesis. It is possible that at different stages (time-points) of unloading the regulation of ribosome biogenesis (c-myc, 18S and 28S rRNA) could depend on different regulatory signaling pathways. L-NAME administration revealed that the PMS effect on the markers of ribosome biogenesis was associated with the NO-related mechanism.

## Conclusion

Thus, the application of PMS during 7-day HS mitigates slow-twitch fiber atrophy and attenuates a decrease in the rate of protein synthesis and the markers of translational efficiency (p70S6k, p90RSK, eEF2) and capacity (18S + 28S rRNA, 45S-pre rRNA, c-myc) in rat soleus muscle. The effects of PMS on muscle protein synthesis and a number of the key anabolic markers were mediated through NO-dependent mechanisms.

## Supplementary Information

Supplementary Information.

## Abbreviations

PMS: Plantar mechanical stimulation
L-NAME: L-N^G^-Nitro arginine methyl ester (L-NAME)
HS: Hindlimb suspension
HU: Hindlimb unloading
PBS: Phosphate buffered saline
RT-PCR: Reverse transcription polymerase chain reaction
p70S6k: Ribosomal protein S6 kinase beta-1 (S6K1)
4E-BP1: Eukaryotic translation initiation factor 4E-binding protein 1
eIF2B: Eukaryotic translation initiation factor 2B
eEF-2: Eukaryotic elongation factor 2
p90RSK: 90 KDa ribosomal s6 kinases
PKB (AKT): Protein kinase B
mTORC1: Mammalian target of rapamycin complex 1
MuRF-1: Muscle RING-finger protein-1
GAPDH: Glyceraldehyde-3-phosphate dehydrogenase
IRS-1: Insulin receptor substrate 1
ERK1/2: Extracellular signal-regulated kinase 1/2
IGF-1: Insulin-like growth factor 1
GSK3beta: Glycogen synthase kinase 3 beta
NOS: Nitric oxide synthase
MyHC: Myosin heavy chain
NFAT: Nuclear factor of activated T-cells
AMPK: AMP-activated protein kinase
CSA: Cross-sectional area
SUnSET: Surface sensing of translation
ROS: Reactive oxygen species
