# Supplementary material for: Plantar mechanical stimulation attenuates protein synthesis decline in disused skeletal muscle via modulation of nitric oxide level
Source: Sci Rep. 2021 May 7;11:9806. doi: 10.1038/s41598-021-89362-6 (PMC8105341; doi:10.1038/s41598-021-89362-6)
Supplement: Supplementary file 1 — Supplementary Information. [file 41598_2021_89362_MOESM1_ESM.pdf]

Plantar mechanical stimulation attenuates protein synthesis decline in disused skeletal muscle via modulation of nitric oxide level

Sergey Tyganov[1], Ekaterina Mochalova[1], Svetlana Belova[1], Kristina Sharlo[1], Sergey Rozhkov[1], Vitaliy Kalashnikov[1], Olga Turtikova[1], Timur Mirzoev[1], Boris Shenkman[1]

[1] Myology Laboratory, Institute of Biomedical Problems RAS, Moscow, Russia

Correspondence: Sergey A. Tyganov, PhD Institute of Biomedical Problems RAS Myology Laboratory 123007, Khoroshevskoe shosse 76a Moscow, Russian Federation, e-mail: sentackle@yandex.ru, tel: +7 985 873 99 01

Running title: Plantar stimulation attenuates protein synthesis decline in NO-dependent manner

Funding: The Russian Foundation for Basic Research grants № 17-29-01029 and 19-015-00089, The Program of Basic Research of the IBMP RAS.

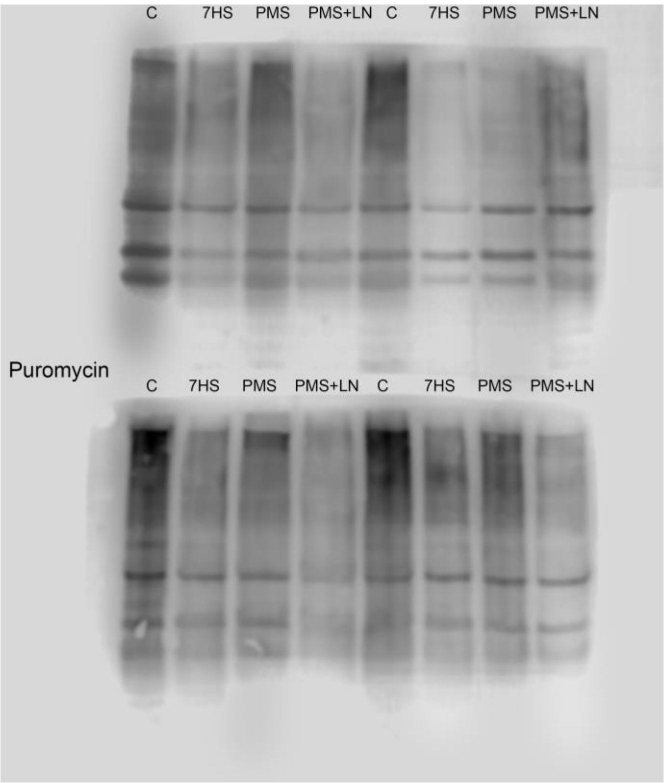
C 7HS PMS PMS+LN C 7HS PMS PMS+LN
Puromycin
C 7HS PMS PMS+LN C 7HS PMS PMS+LN

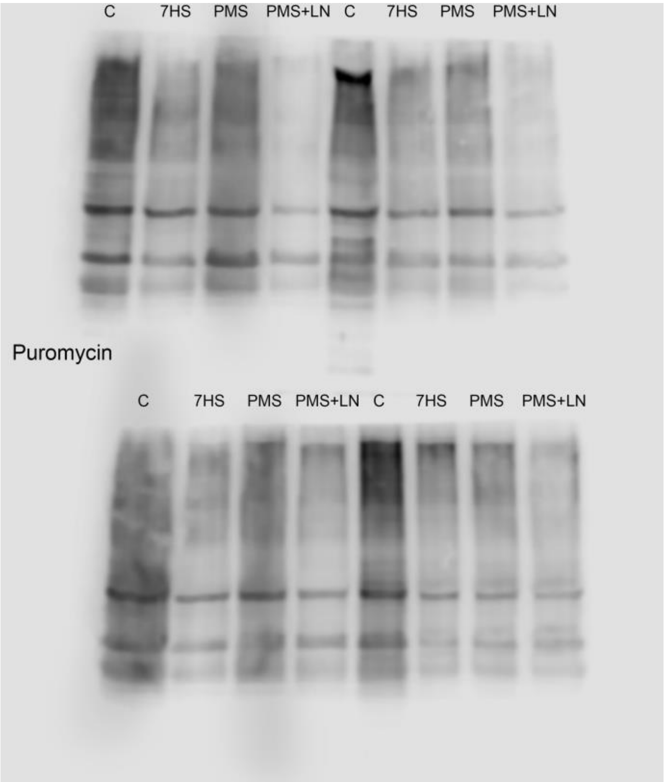
C 7HS PMS PMS+LN C 7HS PMS PMS+LN
Puromycin
C 7HS PMS PMS+LN C 7HS PMS PMS+LN

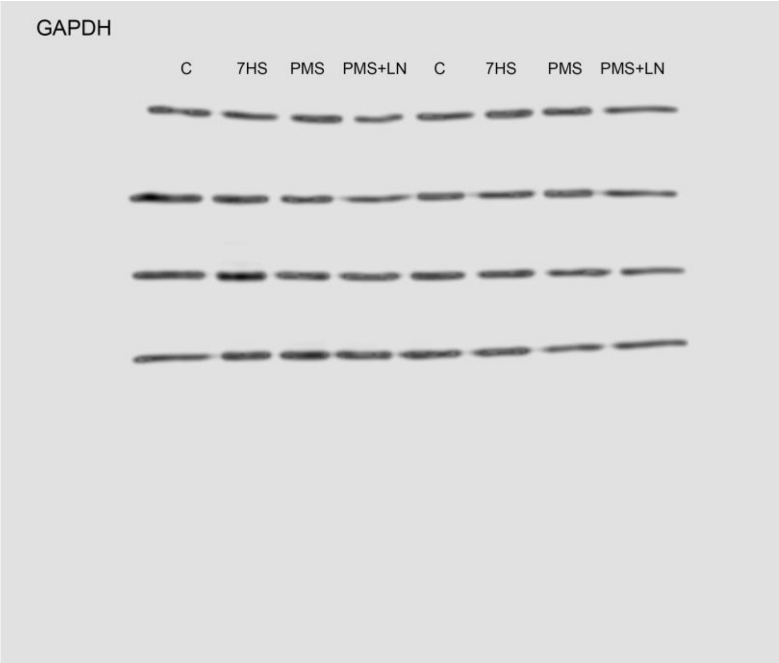
GAPDH
C
7HS
PMS
PMS+LN
C
7HS
PMS
PMS+LN

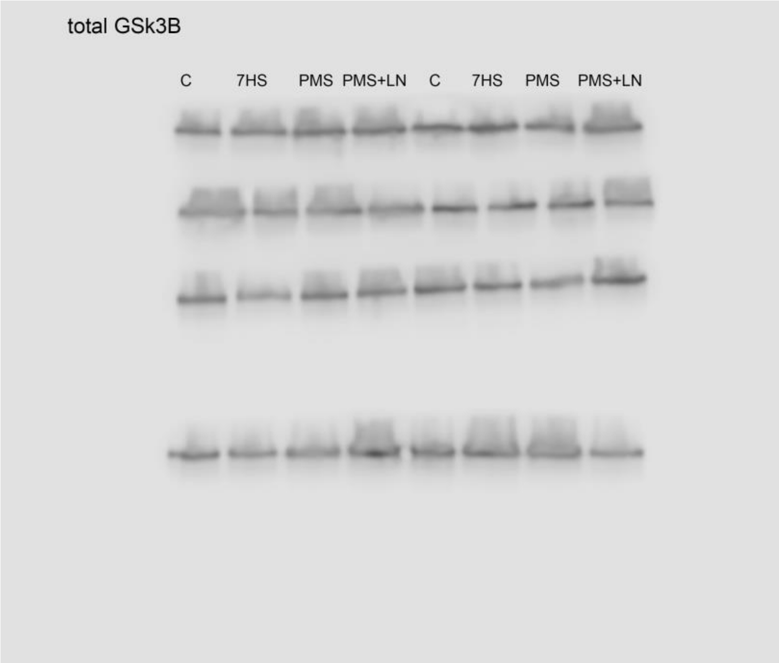
total GSk3B
C
7HS
PMS
PMS+LN
C
7HS
PMS
PMS+LN

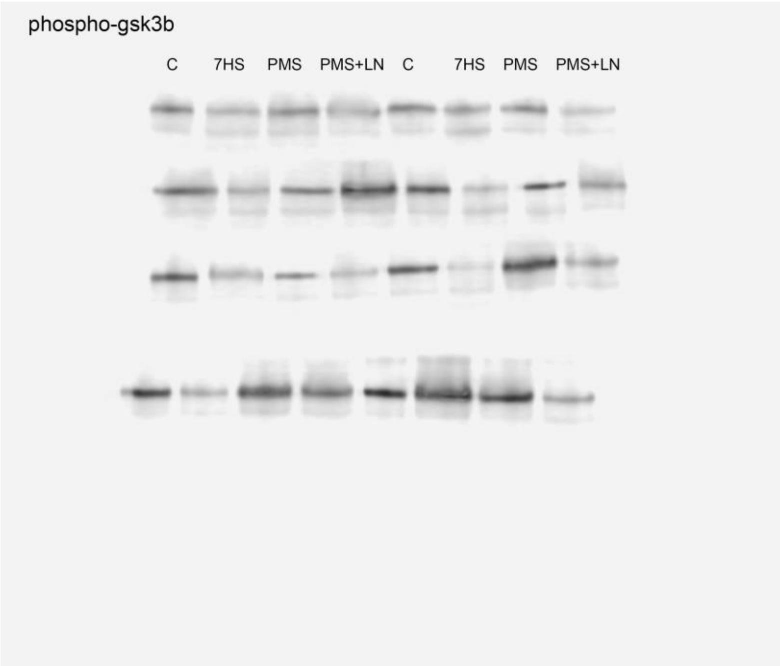
phospho-gsk3b
C
7HS
PMS
PMS+LN
C
7HS
PMS
PMS+LN

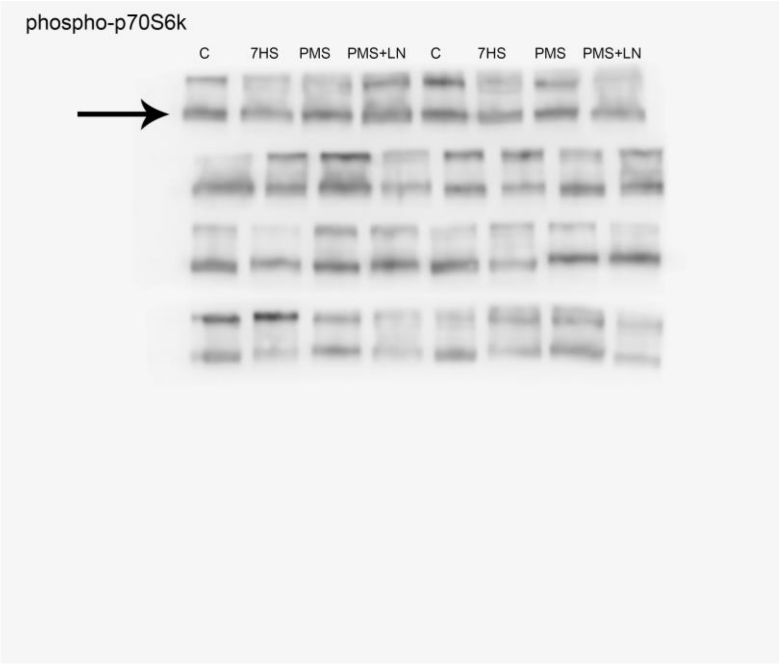
phospho-p70S6k
C 7HS PMS PMS+LN C 7HS PMS PMS+LN

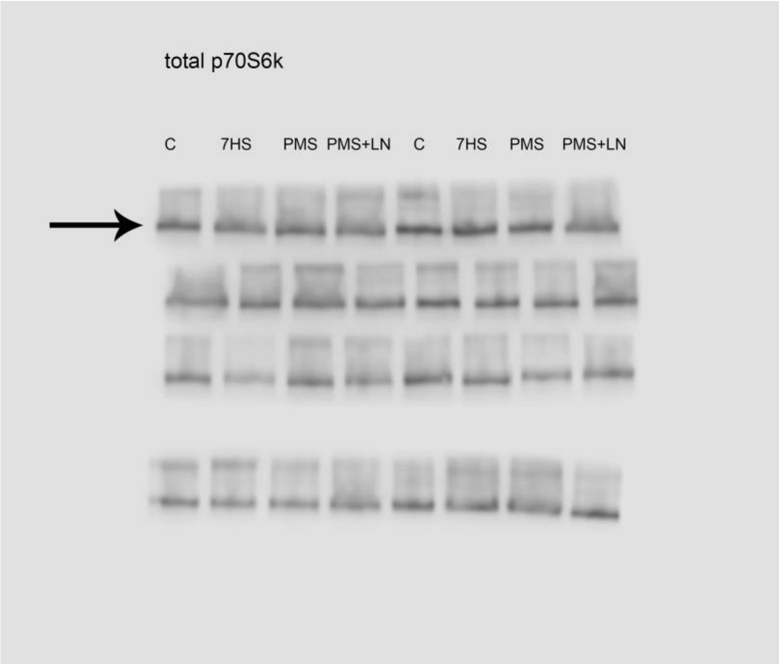
total p70S6k
C 7HS PMS PMS+LN C 7HS PMS PMS+LN

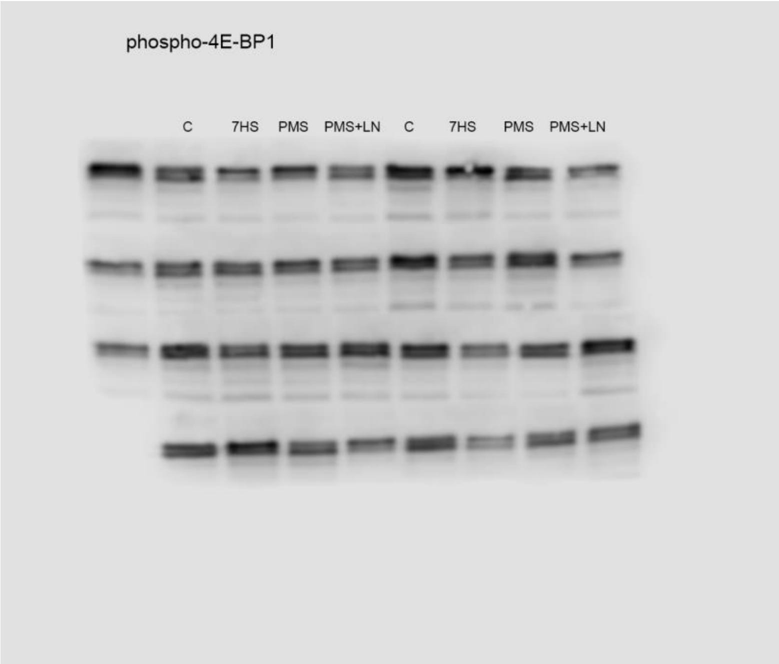
phospho-4E-BP1
C
7HS
PMS
PMS+LN
C
7HS
PMS
PMS+LN

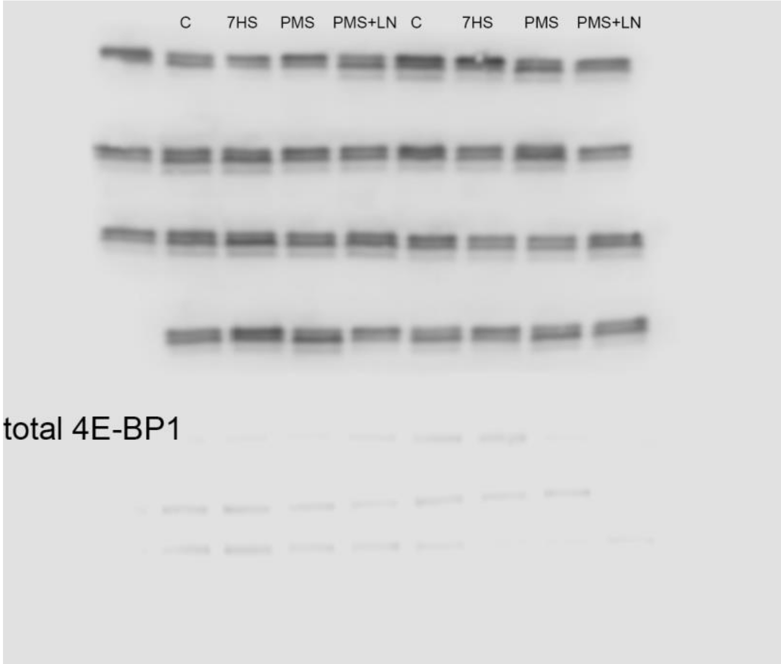
C
7HS
PMS
PMS+LN
C
7HS
PMS
PMS+LN
total 4E-BP1

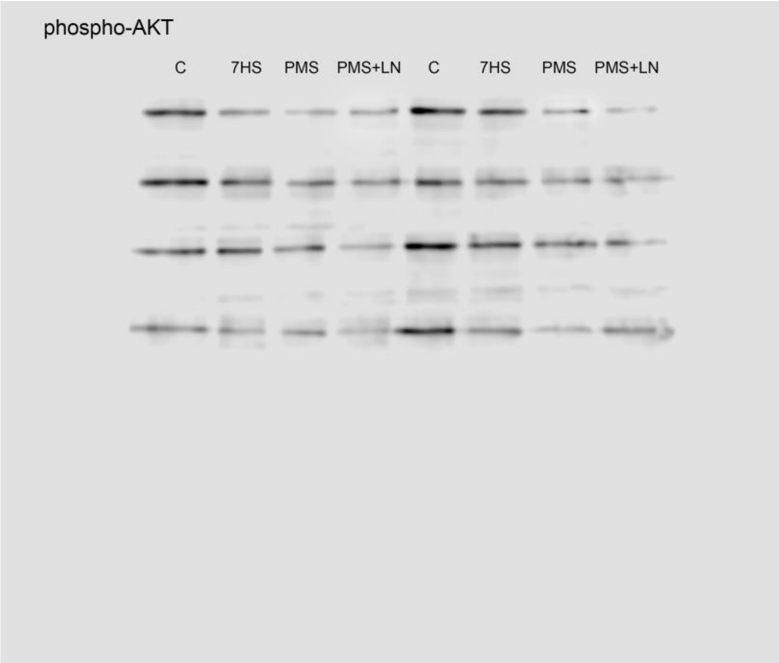
phospho-AKT
C
7HS
PMS
PMS+LN
C
7HS
PMS
PMS+LN

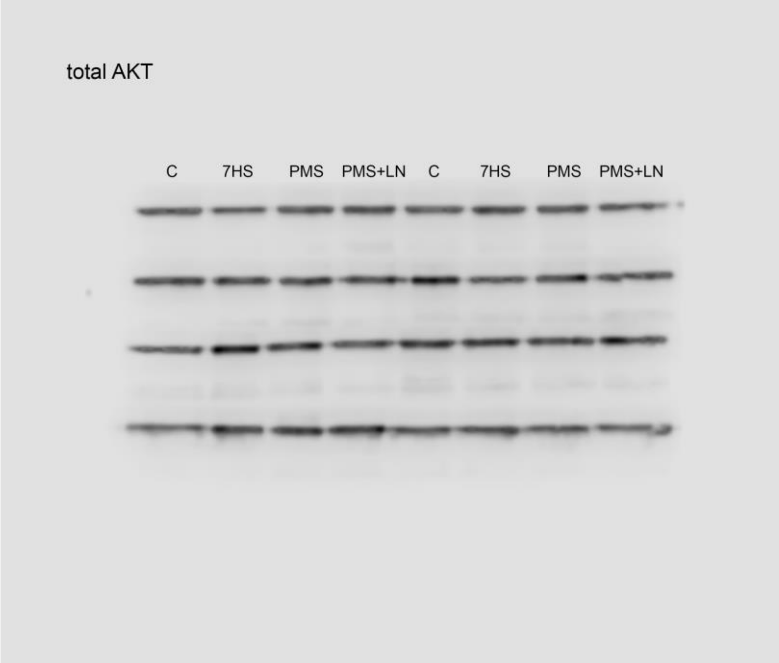
total AKT
C
7HS
PMS
PMS+LN
C
7HS
PMS
PMS+LN

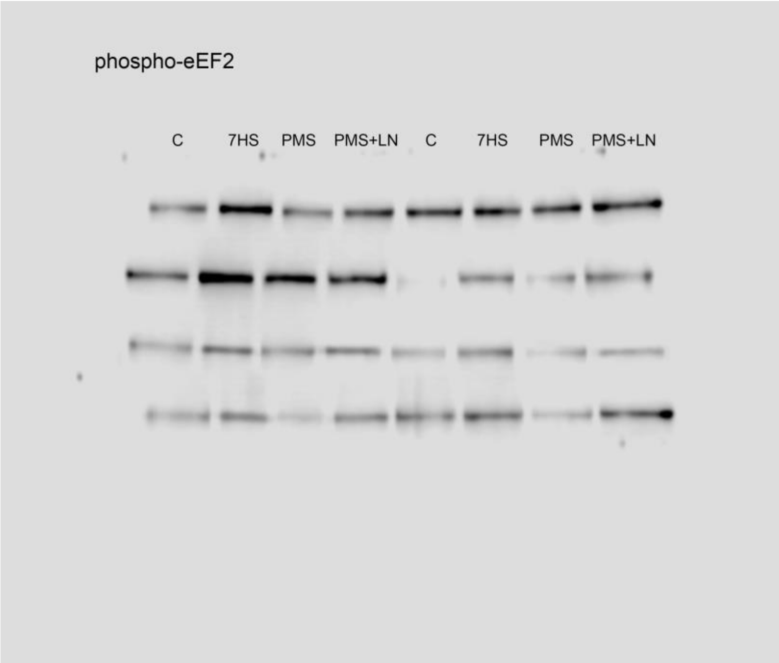
phospho-eEF2
C
7HS
PMS
PMS+LN
C
7HS
PMS
PMS+LN

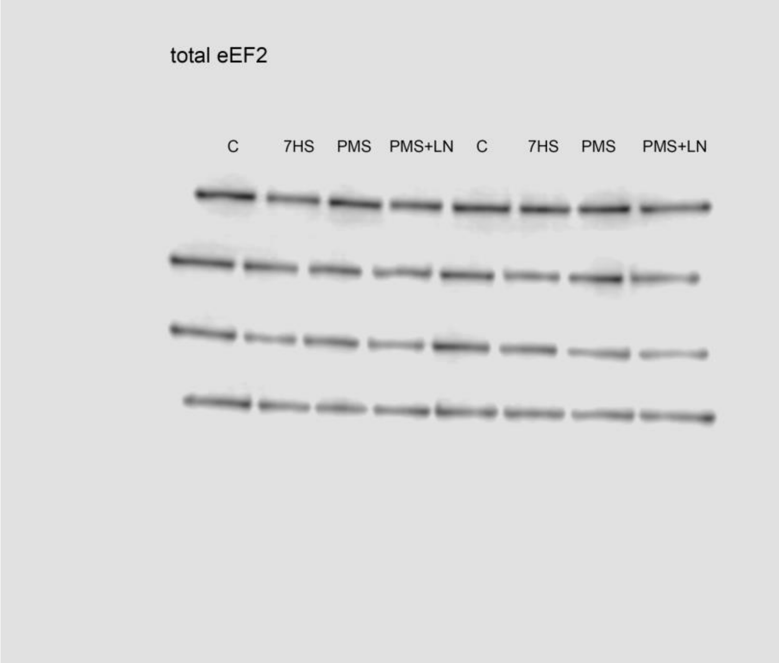
total eEF2
C
7HS
PMS
PMS+LN
C
7HS
PMS
PMS+LN

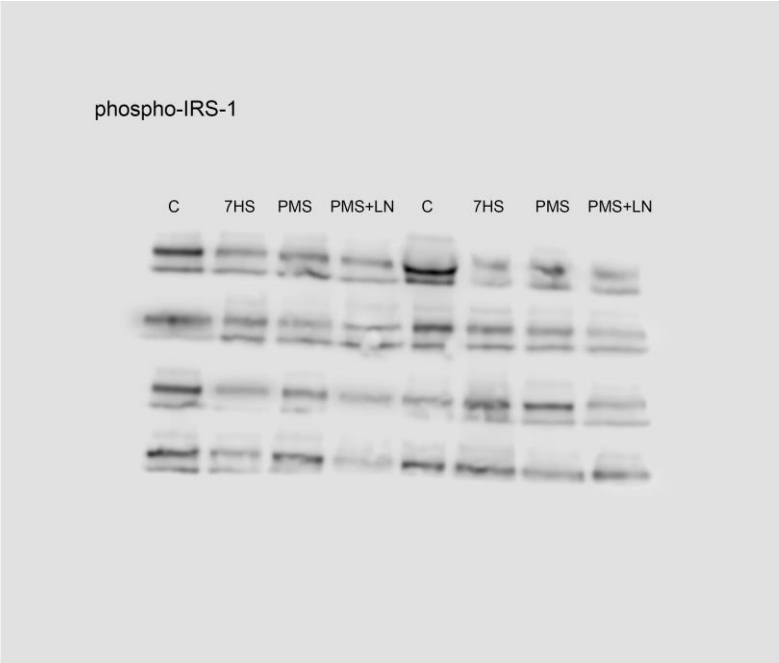
phospho-IRS-1
C 7HS PMS PMS+LN C 7HS PMS PMS+LN

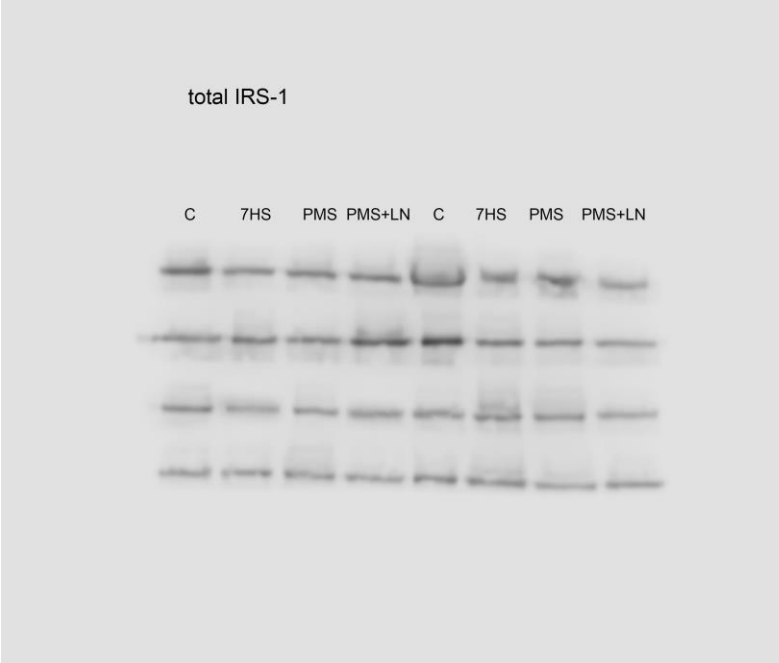
total IRS-1
C 7HS PMS PMS+LN C 7HS PMS PMS+LN

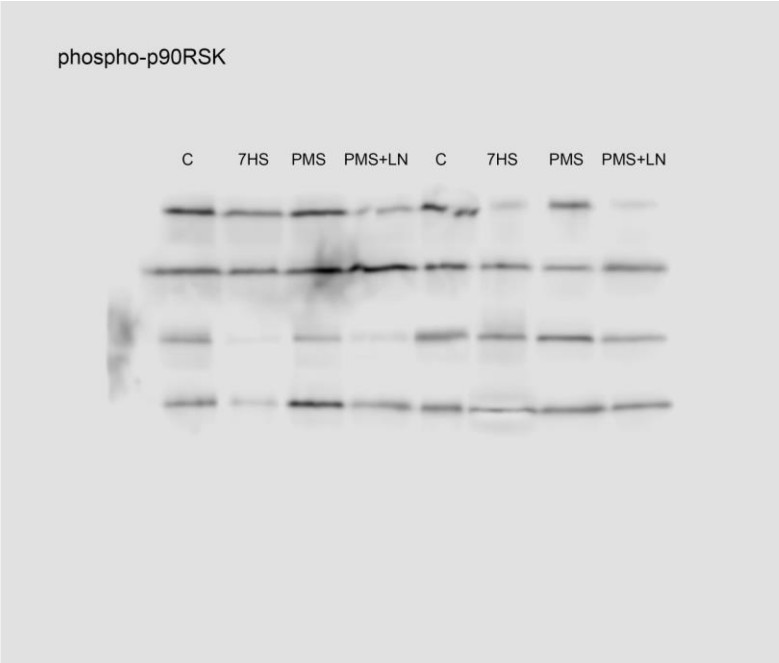
phospho-p90RSK
C 7HS PMS PMS+LN C 7HS PMS PMS+LN

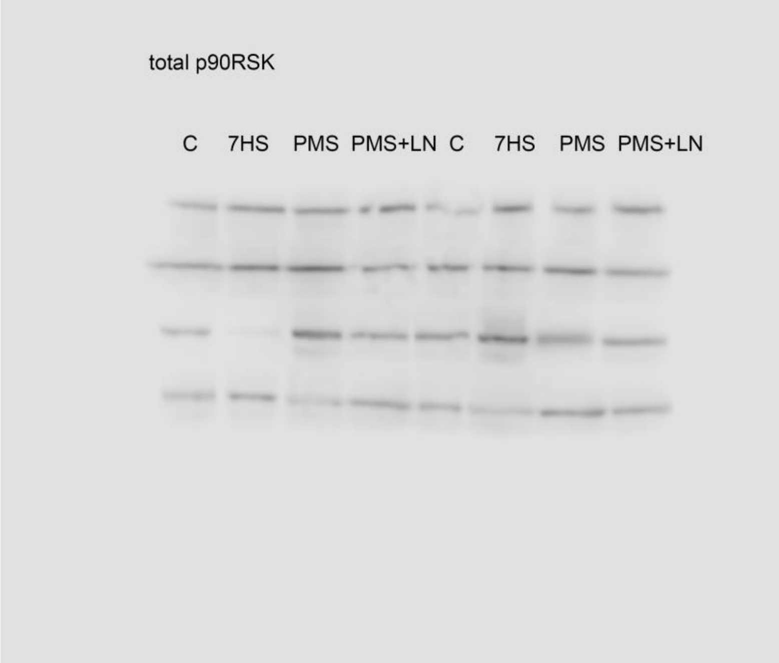
total p90RSK
C 7HS PMS PMS+LN C 7HS PMS PMS+LN
